# Double Proton Tautomerism via Intra- or Intermolecular Pathways? The Case of Tetramethyl Reductic Acid Studied by Dynamic NMR: Hydrogen Bond Association, Solvent and Kinetic H/D Isotope Effects

**DOI:** 10.3390/molecules26144373

**Published:** 2021-07-20

**Authors:** Hans-Heinrich Limbach, Simone Baumgärtner, Roland Franke, Ferdinand Männle, Gerd Scherer, Gleb S. Denisov

**Affiliations:** 1 Institut für Chemie und Biochemie, Freie Universität Berlin, Takustr. 3, 14195 Berlin, Germany; ferdi@klingelberg.no (F.M.); oli4g.scherer@gmail.com (G.S.); 2 Institut für Physikalische Chemie, Universität Freiburg, Albertstr. 21, 79104 Freiburg im Breisgau, Germany; baumgaertner.simone@web.de (S.B.); rfranke@thornebiomed.com (R.F.); 3 GKS Müllheim, Nußbaumallee 6, 79379 Müllheim, Germany; 4 Klingelberg Products AS, Nordåsveien 23, 1415 Oppegård, Norway; 5 Intertek AG, Tech Center Reinach, Kägenstrasse 18, CH-4153 Reinach, Switzerland; 6 Department of Physics, St. Petersburg State University, 198504 St. Petersburg, Russia; gldenisov@yandex.ru

**Keywords:** dynamic NMR spectroscopy, tetramethyl reductic acid, dimer-monomer equilibrium, tautomerization, double proton transfer, kinetic HH/HD isotope effects, tunneling

## Abstract

Using dynamic liquid-state NMR spectroscopy a degenerate double proton tautomerism was detected in tetramethyl reductic acid (TMRA) dissolved in toluene-*d*_8_ and in CD_2_Cl_2_. Similar to vitamin C, TMRA belongs to the class of reductones of biologically important compounds. The tautomerism involves an intramolecular HH transfer that interconverts the peripheric and the central positions of the two OH groups. It is slow in the NMR time scale around 200 K and fast at room temperature. Pseudo-first-order rate constants of the HH transfer and of the HD transfer after suitable deuteration were obtained by line shape analyses. Interestingly, the chemical shifts were found to be temperature dependent carrying information about an equilibrium between a hydrogen bonded dimer and a monomer forming two weak intramolecular hydrogen bonds. The structures of the monomer and the dimer are discussed. The latter may consist of several rapidly interconverting hydrogen-bonded associates. A way was found to obtain the enthalpies and entropies of dissociation, which allowed us to convert the pseudo-first-order rate constants of the reaction mixture into first-order rate constants of the tautomerization of the monomer. Surprisingly, these intrinsic rate constants were the same for toluene-*d*_8_ and CD_2_Cl_2_, but in the latter solvent more monomer is formed. This finding is attributed to the dipole moment of the TMRA monomer, compensated in the dimer, and to the larger dielectric constant of CD_2_Cl_2_. Within the margin of error, the kinetic HH/HD isotope effects were found to be of the order of 3 but independent of temperature. That finding indicates a stepwise HH transfer involving a tunnel mechanism along a double barrier pathway. The Arrhenius curves were described in terms of the Bell–Limbach tunneling model.

## 1. Introduction

**Figure 1:**
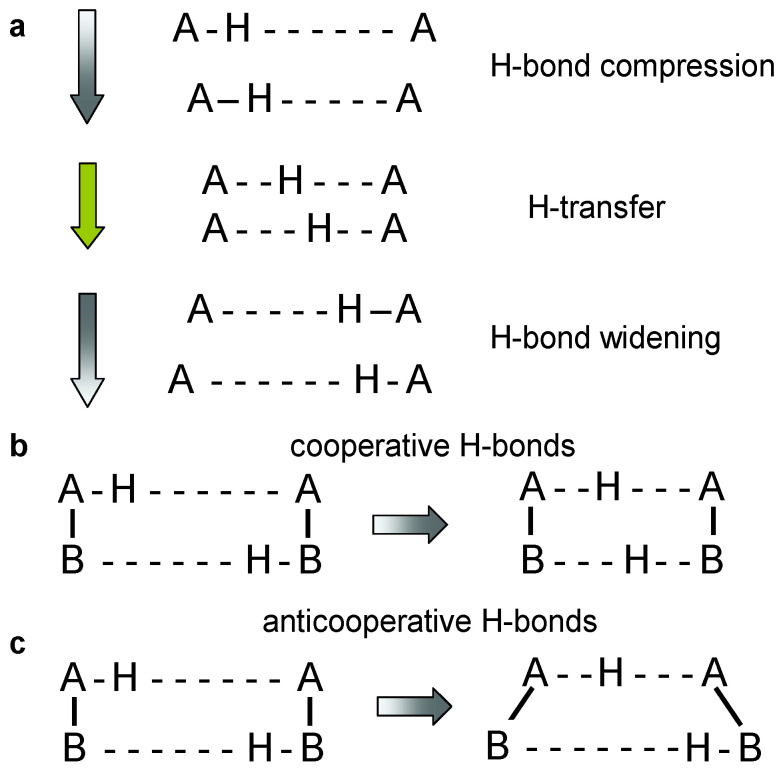
(**a**) Hydrogen bond compression during hydrogen transfer [2,3,4,5,6,7,8,9,10,11,12,13,14,15,16]. (**b**) Cooperativity and (**c**) anti-cooperativity of two coupled hydrogen bonds [5,6,7,8,9,10,11,12]. Cooperativity leads to a concerted double proton transfer, anti-cooperativity to a stepwise double proton transfer.

The interconversion of tautomers of molecules exhibiting two or more proton binding sites can take place via intra- or intermolecular proton transfer pathways [1,2]. Dynamic liquid- and solid-state NMR has been the major technique to discover and characterize these phenomena with respect to structure as well as thermodynamic and kinetic properties [3,4,5,6]. The tautomerization pathways depend on the molecular structure, in particular on the ability to form intra- and/or intermolecular hydrogen bonds. In particular the ease of hydrogen bond compression plays a major role as illustrated in Figure 1a [7,8,9,10,11]. Compression decreases the H-bond heavy atom distance and shifts H towards the H-bond center. H-transfer takes then place at almost constant heavy atom distances either via tunneling or over the barrier, or even in a single-well potential. Thus, tautomerizations are faster the shorter are the hydrogen bonds because of the smaller energy needed for compressing the hydrogen bonds.

In multiple proton transfers the cooperativity of hydrogen bonds is another important feature [12]. As illustrated in Figure 1b,c, two coupled hydrogen bonds might be cooperative or anti-cooperative. In the first case, compression of one bond leads to the compression of the other bond, whereas it leads in the second case to a widening. That is often the case in systems with several intramolecular hydrogen bonds as the molecular skeleton does not allow for the compression of both bonds at the same time. The consequence is a stepwise double proton transfers along intramolecular pathways, whereas concerted pathways are possible in intermolecular hydrogen bonds that can be compressed at the same time.

**Figure 2:**
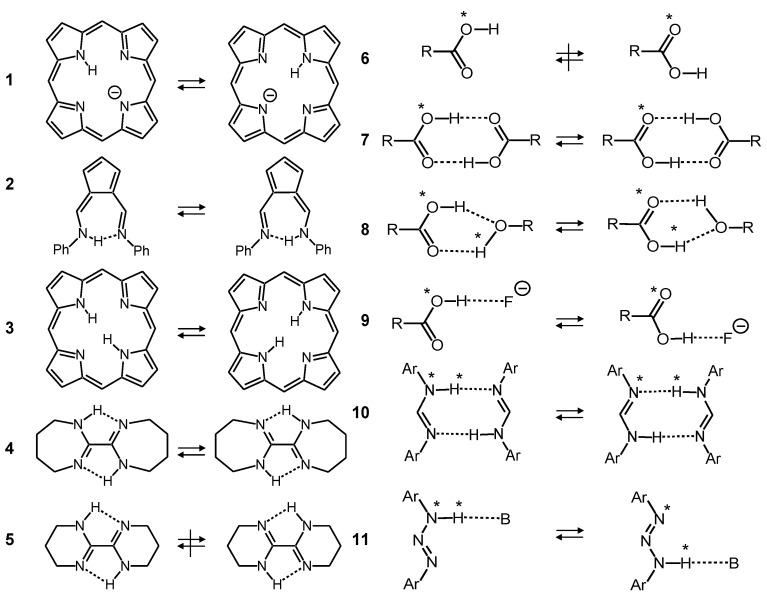
Tautomerism of the porphyrin anion **1** [13], solid *N*,*N*′-diphenyl-6-aminofulvene-1-aldimine **2** [14], porphyrin **3***,* bicyclic oxalamidines **4** and **5** [15,16], carboxylic acid monomer **6**, carboxylic acid dimer **7** [17,18], acetic acid/methanol **8** [19], acetic acid/fluoride **9** [20], *N*,*N*′-diarylamidines **10** [21,22,23] and *N*,*N*′-diaryltriazene/triethylamine **11** [24].

Some examples studied in our laboratory using dynamic liquid- and solid-state NMR spectroscopy [2,5,6] are used to illustrate these mechanisms. The single proton transfer rates of the porphyrin anion **1** [13] (Figure 2), which exhibits very weak intramolecular hydrogen bonds, are therefore smaller than those of the strong hydrogen bond of the fulvenaldimine **2** [14]. Similar effects are observed for intramolecular double proton transfers in porphyrin **3** and related compounds, or the bicyclo-oxalamidines **4**. The kinetic isotope effects indicate stepwise double proton transfers via zwitterionic intermediates, requiring the successive hydrogen bond compression of the two rings for the tautomerization to occur [15,16]. In the case of the oxalamidine, this is possible for the flexible 7-membered rings of **4**, whereas the rigidity of the 6-membered rings of **5** leads to a quench of the tautomerization. We note that in the resonance-assisted tautomerizations of **1** to **4** small displacements of the atoms of the molecular skeletons occur during the H-transfer, which increase the tunneling masses [5,6].

However, there are many molecules which can adopt different tautomers but do not exhibit intramolecular hydrogen bonds. That is the case of carboxylic acid monomers **6**. Thus, intramolecular transfer from one oxygen to the other is quenched under normal conditions. However, the mobile protons can usually form strong intermolecular hydrogen bonds with other proton donors or acceptors. Thus, the formation of cyclic dimers **7** enables very fast double proton transfers in the solid state [17,18] and in liquid solution [19]. Additionally, other proton donors can catalyze the tautomerization of carboxylic acids as, for example, methanol, which forms in aprotic solvents a cyclic 1:1 complex **8** with acetic acid [19]. Moreover, a simple base such as a fluoride anion can carry the proton from one oxygen atom of acetic acid to the other **9** [20]. Related processes have been studied in the case of diarylamidines, which represent the nitrogen analogs of carboxylic acids. They form cyclic dimers **10** in which fast double proton transfers take place in the liquid [21,22] and in the solid state [23]. X-ray crystallography indicates that the aryl groups are outside the molecular plane, which enables the formation of the cyclic dimers. By contrast, in the related diaryltriazenes **11** the aryl groups are located in the molecular plane, and cyclic dimers cannot be formed [24]. However, organic bases such as trimethylamine can form hydrogen bonds and carry the proton from one nitrogen atom to the other. That process constitutes an intramolecular catalyzed tautomerization.

**Figure 3:**
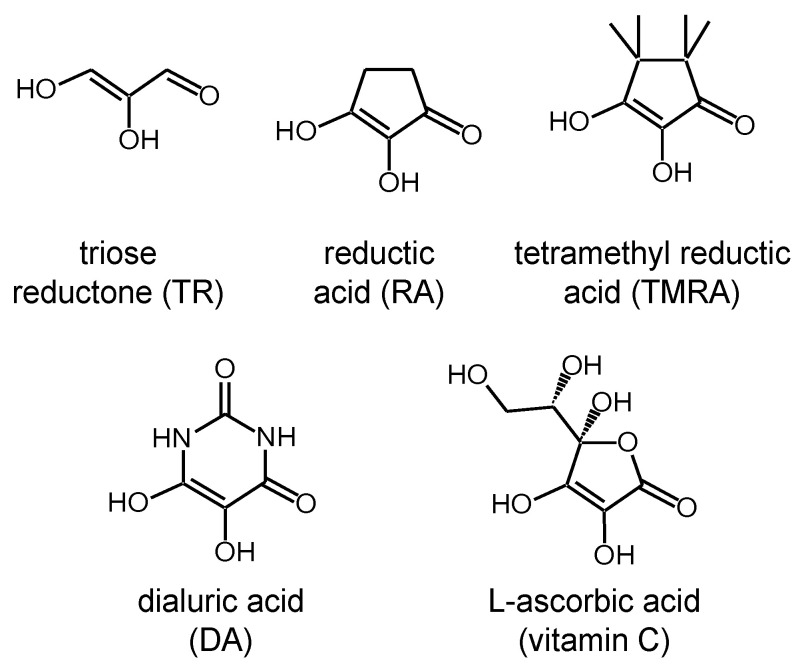
A selection of reductones. The present NMR study deals with the tautomerization of tetramethyl reductic acid (TMRA) in CD_2_Cl_2_ and toluene-*d*_8_.

So far, to our knowledge, there is little known about the tautomerism of molecules that can potentially form both intra- as well as intermolecular hydrogen bonds. The question arises at which point the switch from intramolecular to intermolecular proton transfer occurs. In our search for suitable model systems to study this problem, the attention fell on the class of the biologically important reductones depicted in Figure 3 [25,26]. These molecules are able to reduce organic compounds while being oxidized at the same time. Reductones contain two OH groups bound to a carbon double bond adjacent to a C=O group. The simplest representants are triose reductone (TR), reductic acid (RA) and tetramethyl reductic acid (TMRA). They represent analogues of biologically important molecules such as dialuric acid (DA) and L-ascorbic acid (vitamin C) [27,28,29,30].

**Figure 4:**
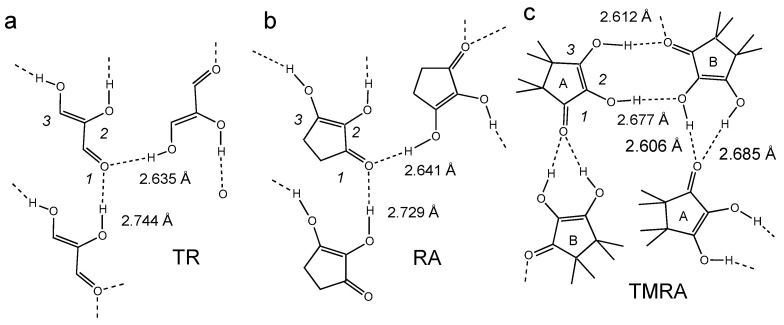
Hydrogen bond patterns (schematically) resulting from the crystallographic structures of (**a**) triose reductone TR [31], (**b**) reductic acid RA [32] and (**c**) TMRA [33]. The O…O distances of the OHO hydrogen bonds are included.

The hydrogen bond patterns in the crystalline states of the TR, RA and TMRA are depicted schematically in Figure 4. In TR and RA, the two OH groups form hydrogen bonds with the C=O groups of two neighboring molecules [31,32]. In crystalline TMRA there are two inequivalent molecules, A and B [33]. The two OH groups of B form two hydrogen bonds with the C=O group of molecule A. The central OH group of the latter forms a hydrogen bond with the central OH group of molecule B, and the peripheric group with the C=O group of B. The O…O hydrogen bond distances in all cases are around 2.6–2.7 Å.

In the solid state the degenerate proton tautomerizations of the reductones TR, RA and TMRA are quenched. However, that may not be the case when they are dissolved in organic solvents. Therefore, we became interested in 1984 and the following years to explore the tautomerism and the hydrogen bond situation of symmetrical reductones using dynamic NMR spectroscopy. To our knowledge, these phenomena have not been reported until today. As triose reductone and reductic acid are subject to a slow keto-enol tautomerism that could complicate the data analysis, we chose to study in this work TMRA, which is unable to form a keto tautomer [34].

**Figure 5:**
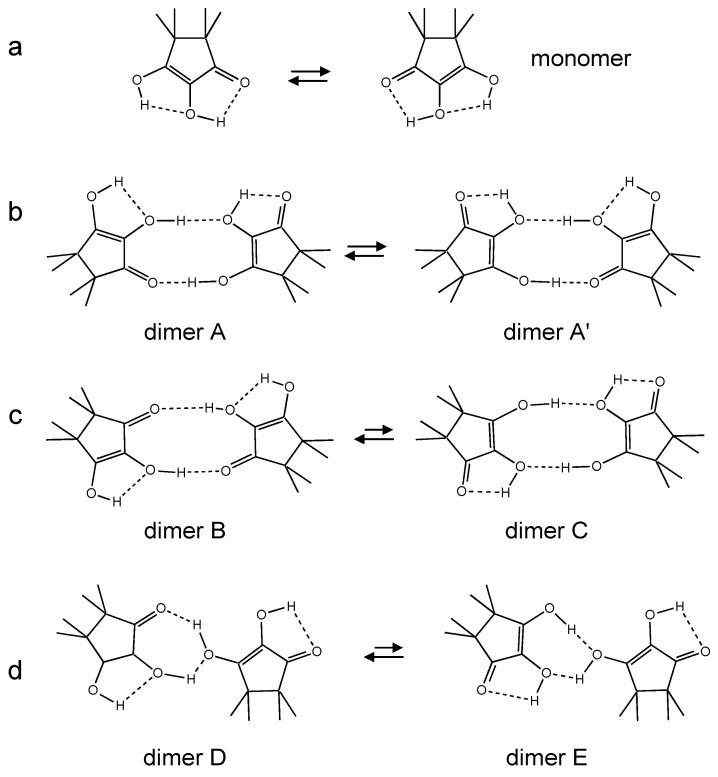
Potential intra- and intermolecular proton tautomerizations of TMRA in organic solvents. (**a**) Degenerate HH transfer of TMRA monomers. (**b**) Degenerate inter/intramolecular HHHH transfer in dimer A. (**c**) Non-degenerate inter/intramolecular HHHH transfer between dimers B and C. (**d**) Non-degenerate inter/intramolecular HHH transfer between dimers D and E.

In Figure 5 some possible hydrogen bonded structures of TMRA are depicted as one may expect for liquid solutions. Monomeric TMRA exhibits two weak intramolecular hydrogen bonds and possibly a degenerate intramolecular double proton (HH) transfer (Figure 5a). Structures of dimers of TMRA can be derived assuming that both OH groups are involved either in inter- or intramolecular hydrogen bonds. Dimer A exhibits a cyclic structure with an intermolecular O-H···O=C, an intermolecular OH···OH and two intramolecular OH···OH hydrogen bonds (Figure 5b) enabling a degenerate tautomerization to dimer A’. This tautomerism involves the transfer of four protons (HHHH), two along an intermolecular and two along an intramolecular pathway. Dimer B exhibits a non-degenerate HHHH transfer leading to dimer C (Figure 5c). Finally, dimer D also may be present, which could be converted via a non-degenerate HHH transfer into dimer E. It is difficult to say which structure dominates in the last two cases. The monomer and all dimers interconvert rapidly in solution as hydrogen bond exchange is much faster than multiple proton transfers. Note, however, that only the latter leads to an exchange of the two OH protons.

In order to explore this very complex behavior we undertook the present NMR studies described in the following. In the Experimental Section, the synthesis of TMRA, the solvent purification and sample preparation of TMRA, the details of the NMR experiments and the data analysis techniques are described. Then, the results of the NMR experiments and the line shape analyses are reported. Two solvents were employed, the polar CD_2_Cl_2_ and the apolar toluene-*d*_8_. Indeed, we found a proton transfer process that averages the signals of the outer and the center OH protons in the NMR time scale. A sample deuterated in the mobile proton sites was prepared in order to explore potential kinetic H/D isotope effects. In the Discussion Section we firstly analyze the temperature-dependent ^1^H chemical shifts of TMRA solutions in terms of a monomer–dimer equilibrium. Finally, the resulting Arrhenius diagrams are discussed. We show that dimerization plays a dominant role in the tautomerism of TMRA, whose mechanism is discussed.

## 2. Materials and Methods

### 2.1. Synthesis of TMRA

The synthesis of TMRA started from phorone (2,6-dimethyl-2,5-heptadien-4-one, Sigma-Aldrich, Taufkirchen, Germany, CAS Reg. No. 504-20-1), which was converted into phorone tetrabromide (2,3,5,6-tetrabromo-2,6-dimethyl-4-heptanone, CAS Reg. No. 73806-71-0) according to Claisen [35]. In the next step, phorone dibromide (3,5-Dibromo-2,6-dimethyl-2,5-heptadien-4-one, CAS Reg. No. 5682-79-1) was synthesized according to Francis et al. [36]. In the last step, the latter was converted into 2,3-dihydroxy-4,4,5,5-tetramethyl-2-cyclopenten-1-one (tetramethyl reductic acid, TMRA, CAS Reg. No. 1889-96-9) using the procedure of Hesse et al. [34].

TMRA easily oxidizes to the hydrate of 4,4,5,5-tetramethyl-1,2,3-cyclopentanetrione (CAS Reg. No. 1889-98-1), which decomposes into green reaction products [37]. Therefore, the compound was recrystallized 3 times from bidistilled water, which gave colorless crystals [34]. Finally, the compound was dried in vacuo.

### 2.2. Sample Preparation

The NMR samples were prepared using vacuum techniques described previously [19,38]. The different sections, consisting of solvent glass vessels, calibrated glass tubes for volume measurements of liquids and the NMR tube could be opened and closed using Teflon needle valves. As NMR tubes, normal 5 mm tubes were employed, which could be separated from the vacuum line by flame sealing or which were equipped with a Teflon Young needle valve (Wilmad, Buena, NJ, USA). The deuterated solvents were stored in glass vessels over a drying agent, attached to the vacuum line and degassed several times. In order to remove moisture from the samples, basic alumina was used for CD_2_Cl_2_ and sodium/potassium alloy/anthracene for for toluene-*d*_8_. Desired quantities of TMRA were weighed, dissolved in ether and poured into the NMR tube using a pipette. The tube was then attached to the vacuum line, degassed at 77 K and the ether removed in vacuo. Then, dry deuterated solvent was condensed under vacuum into the NMR tube and TMRA dissolved. After dissolution and freezing again, the solvent was removed via the gas phase. That procedure allowed us to remove the remaining moisture in the NMR samples. Finally, a desired quantity of the solvent was condensed into the NMR tube, which was then sealed and taken off from the vacuum line.

We report here the NMR results obtained for four samples characterized in Table 1. For sample #1, CD_2_Cl_2_ was used as solvent and for the other samples toluene-*d*_8_. In the case of sample #4, the mobile proton sites were deuterated to about 88%. Samples #2 and #3 differed by their concentrations. The concentrations of samples #1, #2 and #4 were obtained by weighing, whereas the concentrations of sample #3 were obtained by ^1^H NMR as described in Section 2.4.

### 2.3. ^1^H NMR Measurements

The ^1^H NMR measurements were done using a Bruker FT NMR Spectrometer CXP 100 (Bruker, Forchheim, Germany) exhibiting a Larmor frequency of *ν*_o_= 90.02 MHz for ^1^H. The sample temperatures were monitored with a set of thermometers of diameter 5 mm inserted into the NMR rotor. Simple-phase alternating pulse sequences were used with pulse durations between 1.2 and 1.6 μs (90° pulse lengths around 7 μs). Recycle delays were about 3 s. On average, the number of scans was about 1000 for all samples. We did not use sample spinning, which results in rotational sidebands and difficulties in the analyses of exchange broadened line shapes.

### 2.4. Analysis of the NMR Data

The spectra were available in digital form and processed on a PC. The line shape changes of the two OH groups of TMRA were analyzed in terms of a symmetric two-site exchange using the line shape expression of Gutowsky et al. [39]. For that purpose, a MATLAB program was written. Parameters of a given line shape simulation were the two chemical shifts of the OH groups, *ν*_i_ − *ν*_TMS_ = *ν*_o_*δ*_i_, *i* = 1,2, the line widths in the absence of exchange, *W*_oi_ and the pseudo-first order rate constant *k*_ex_/s^−1^ of exchange of the two OH groups.

In order to simulate the OH signal line shapes of partially deuterated samples we needed to know the signal fractions of the HH and the HD species. For a statistical distribution of D among the isotopic species it is easy to show that the HD signal fraction is equal to the hydroxyl deuterium fraction *X*_D_ = (*x*_HD_ + 2*x*_DD_)/2, where *x*_HH_, *x*_HD_ and *x*_DD_ are the isotopic molefractions. The corresponding HH signal fraction is *X*_H_ = 1 − *X*_D_ = (2*x*_HH_ + *x*_HD_)/2 was determined by integration from the ratio *R* of the OH vs. the methyl signal intensity given in view of the 12 methyl protons of TMRA by *R* = (1 − *X*_D_)/6.

The average ^1^H chemical shifts *δ*_A_ of the mobile OH groups of A≡TMRA were analyzed in terms of an equilibrium between a dimer A_2_ and a monomer A

(1) $$A_{2}\overset{K}{⇄}\;2A\;,$$

where *K* is the equilibrium constant in mol L^−1^ given by

(2) $$K=\frac{c_{A1}^{2}}{c_{A2}},\;C_{A}=c_{A1}+2c_{A2}.$$

*c*_A1_ and *c*_A2_ are the concentrations of the monomer and the dimer, and *C*_A_ the total concentration of TMRA. The mole fractions of the monomer and the dimer are given by

(3) $$x_{A1}=\frac{c_{A1}}{C_{A}},\;x_{A2}=\frac{c_{A2}}{C_{A}},\;\mathrm{with}\;x_{A1}+\;2x_{A2}=1.$$

The temperature dependence of *K* is given by the van’t Hoff equation

(4) $$K=\frac{c_{A1}^{2}}{c_{A2}}=C_{A}\frac{x_{A1}^{2}}{x_{A2}}=\exp(-\Delta G/RT)=\exp(-\Delta H/RT+\Delta S/R),$$

where *R* is the gas constant. Δ*G*, Δ*H* and Δ*S* are the free enthalpy, the enthalpy and the entropy of dissociation of the dimer into monomers, respectively. The average chemical shifts of the coalesced OH groups of TMRA are given by

(5) $$\delta_{A}=x_{A1}\delta_{A1}+2x_{A2}\delta_{A2}\Rightarrow \delta_{A}=x_{A1}(\delta_{A1}-\delta_{A2})+\delta_{A2}.$$

*δ*_A1_ and *δ*_A2_ are the averaged chemical shifts of the two OH groups in the monomer and in the dimer, respectively. For the mole fraction of the monomer it follows that

(6) $$x_{A1}=1-2x_{A2}=\frac{\delta_{A}-\delta_{A2}}{\delta_{A1}-\delta_{A2}}.$$

By combination of the above equations we obtain the following expressions as described previously for other monomer–dimer equilibria [21],

(7) $$x_{A1}=1-2x_{A2}=\frac{K}{4C_{A}}\left[\sqrt{\frac{8C_{A}}{K}+1}-1\right]$$

and

(8) $$\delta_{A}=(\delta_{A1}-\delta_{A2})\frac{K}{4C_{A}}\left[\sqrt{\frac{8C_{A}}{K}+1}-1\right]+\delta_{A2}.$$

For *K* = *C*_A_ it follows that *x*_A1_ = 0.5, i.e., half of the molecules are then monomeric and the other half present in the form of dimers.

If we assume a degenerate proton exchange that can take place both in the monomer as in the dimer—which interconvert quickly within the NMR time scale—then the average inverse proton lifetime or pseudo-first-order rate constant is given by [21]

(9) $$k_{\mathrm{ex}}=k_{A1}x_{A1}+2k_{A2}x_{A2}=k_{A2}+x_{A1}(k_{A1}-k_{A2}).$$

Here, *k*_A1_, and *k*_A2_ represent the rate constants of tautomerim in the monomer and in the dimer. It follows that

(10) $$k_{\mathrm{ex}}=k_{A2}+(k_{A1}-k_{A2})\frac{K}{4C_{A}}\left[\sqrt{\frac{8C_{A}}{K}+1}-1\right].$$

In conclusion, at a given temperature, the average OH chemical shift *δ*_A_ depends on the parameters *C*_A_, *δ*_A1_, *δ*_A2_ and *K*, and the exchange rate constants *k*_ex_ on *C*_A_, *k*_A1_, *k*_A2_ and *K.*

**Figure 6:**
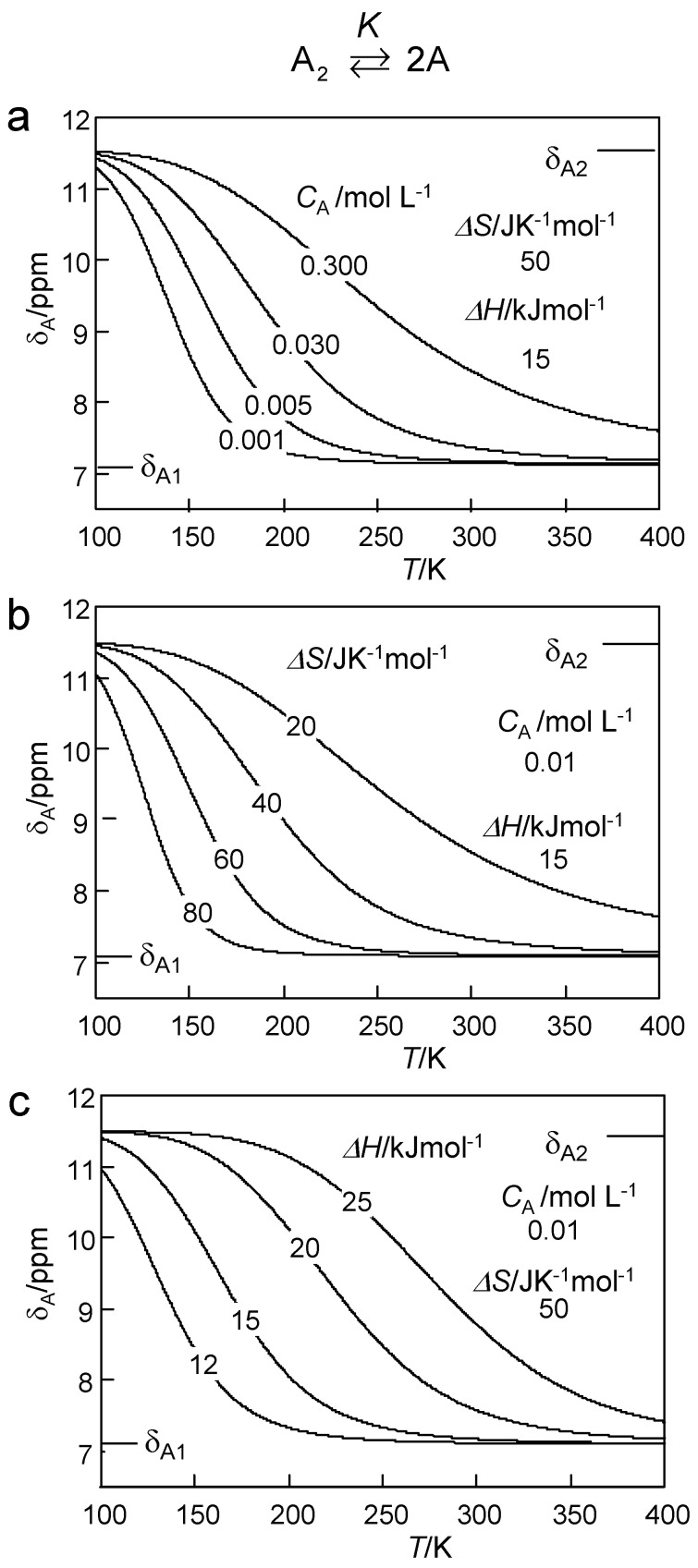
Model calculations according to Equations (4)–(8) of the influence of the dimer–monomer dissociation of a proton donor A on the average chemical shifts δ_A_ of the mobile proton as a function of temperature. δ_A1_ and δ_A2_ are chemical shifts of the monomer and the dimer, respectively. Δ*H* and Δ*S*: reaction enthalpy and entropy of the dimer–monomer dissociation. *C*_A_: total concentration of the proton donor A. (**a**) Variation of *C*_A_, (**b**) variation of Δ*S* and (**c**) variation of Δ*H*. For further explanation see text.

By combining Equations (4) and (8), we were able to explore the influence of various parameters on the chemical shifts *δ*_A_ of a proton donor subject to a dimer–monomer equilibrium according to Equation (1) by writing another MATLAB program. Some results are depicted in Figure 6, where the *δ*_A_ values are plotted as a function of temperature. In each graph only one parameter was varied, i.e., *C*_A_ in Figure 6a, Δ*S* in Figure 6b and Δ*H* in Figure 6c. The curves in Figure 6a are almost identical with those of Figure 6b. That means, that it is not possible to determine both *C*_A_ and Δ*S* for a given sample, i.e., *C*_A_ must be known in order to determine Δ*S*. By contrast, changing Δ*H* leads to different slopes in the plots of *δ*_A_ versus the inverse temperature. Thus, Δ*H* can be obtained even without knowledge of *C*_A_.

## 3. Results

### 3.1. ^1^H NMR Spectroscopy

**Figure 7:**
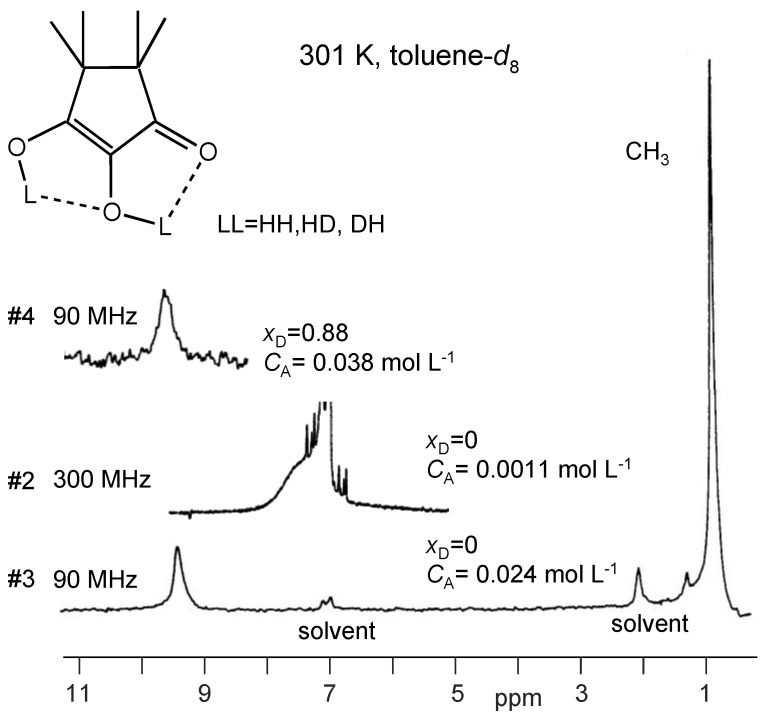
^1^H NMR spectra of TMRA at 301 K dissolved in toluene-*d*_8_ at different concentrations. The concentration of sample #3 was determined as shown in Section 3.2.

In Figure 7 are depicted the partial 90 MHz ^1^H NMR spectra of TMRA in toluene-*d*_8_ solution at 301 K. The residual signals of the solvent resonate at 7.0 and 2.03 ppm. The methyl signal of TMRA is observed at 0.85 ppm. A relatively sharp OH signal appears for sample #3 at 9.34 ppm, whereas the residual OH protons of the deuterated sample #4 resonate at 9.63 ppm. A deuteron fraction of *X*_D_ = 0.88 was obtained from the ratio of the integrated methyl and OH signal intensities (see Section 2.4). The spectrum of the diluted sample #2 was obtained at 300 MHz, where the OH signal is observed at 7.4 ppm. The line width is larger than in the spectra of the other samples because of the larger Larmor frequency.

**Figure 8:**
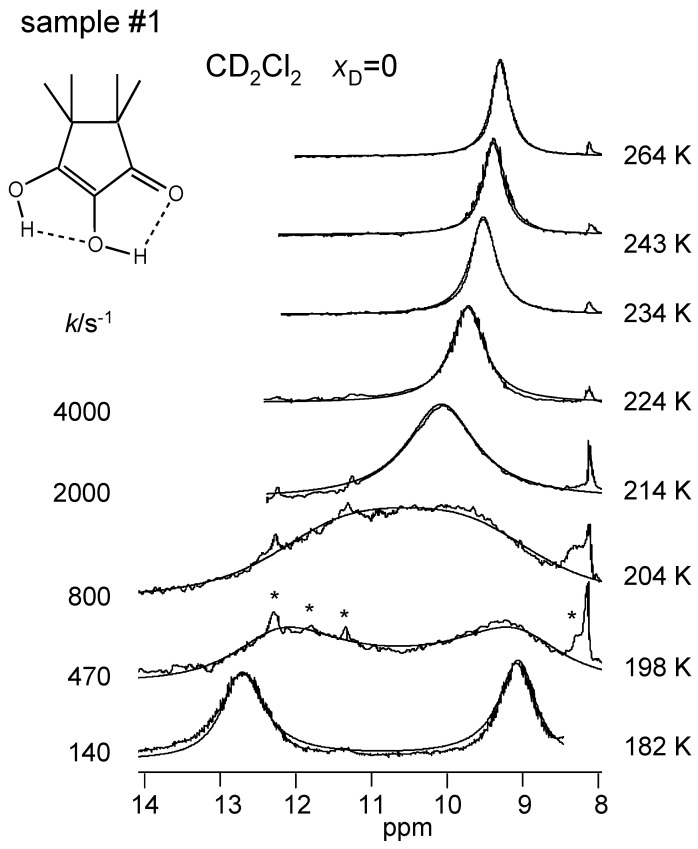
Variable temperature superimposed experimental and simulated ^1^H NMR signals of the hydroxyl groups of TMRA dissolved in CD_2_Cl_2_ (sample #1). The simulation parameters are assembled in Table 2. The asterisk indicates the presence of an unidentified TMRA decomposition product. For further explanation see text.

**Figure 9:**
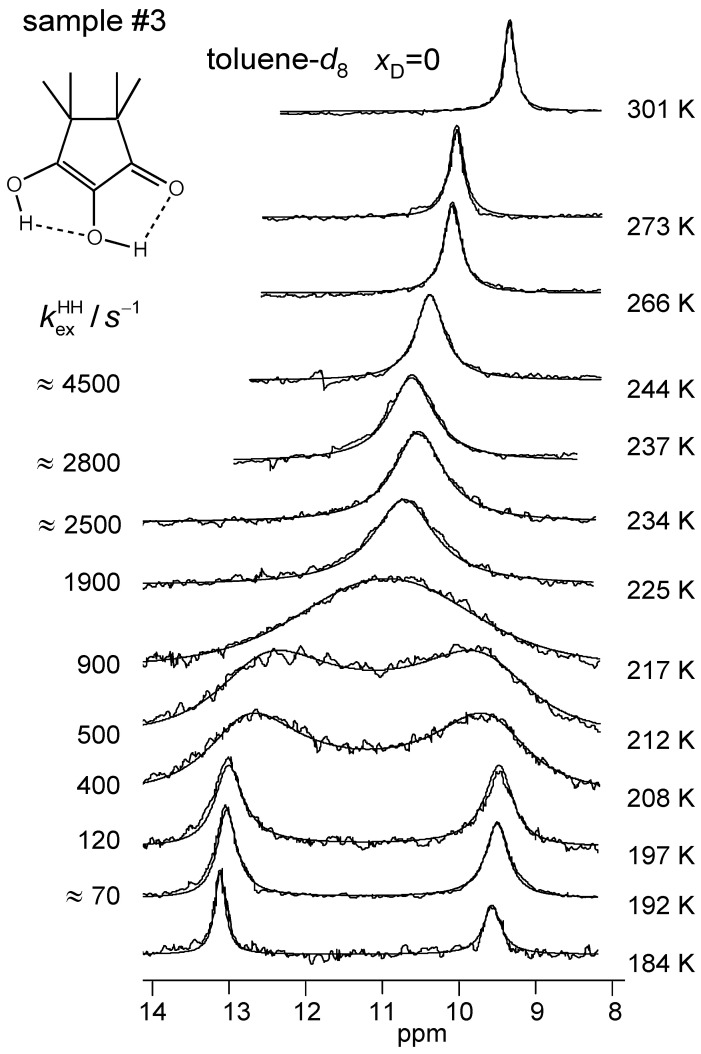
Variable temperature superimposed experimental and simulated ^1^H NMR signals of the hydroxyl groups of TMRA dissolved in toluene-*d*_8_ (sample #3). The simulation parameters are assembled in Table 2. For further explanation see text.

**Figure 10:**
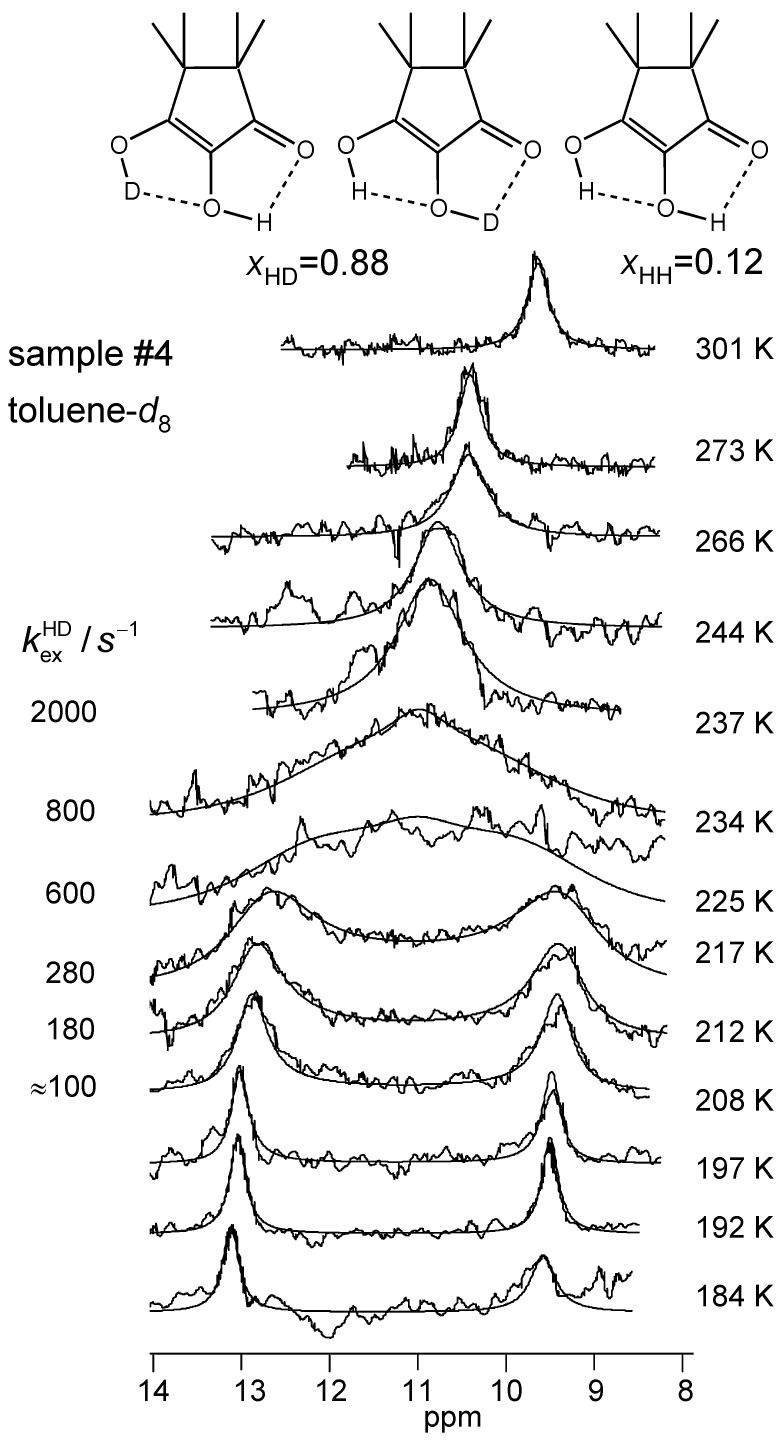
Variable temperature superimposed experimental and simulated ^1^H NMR signals of the partially deuterated hydroxyl groups of TMRA dissolved in toluene-*d*_8_ (sample #4, deuterium fraction *X*_D_ = 0.88, see Section 2.4). The simulation parameters are assembled in Table 2. For further explanation see text.

The superimposed experimental and calculated variable temperature ^1^H NMR spectra of samples #1, #3 and #4 are depicted in Figure 8, Figure 9 and Figure 10. Corresponding spectra of the dilute sample #2 could not be obtained. At low temperatures, the outer and the inner OH groups exhibit different chemical shifts giving rise to two singlets. These signals broaden and coalesce upon increasing temperature, which indicates an averaging of the chemical shifts of both OH protons via proton transfer. At the same time, the signals are shifted to high field. The reaction is faster in the CD_2_Cl_2_ sample #1 than in the toluene-*d*_8_ sample #3 as visualized by the different coalescence temperatures. The signals of the deuterated sample #4 stem to 88% from TMRA-HD and 12% from TMRA-HH. The coalescence temperature is higher, indicating that the tautomerization of the isotopomer TMRA-HD is slower than of TMRA-HH. The chemical shifts of samples #3 and #4 were the same in the slow-exchange regime, whereas at high temperature the small chemical shift difference of both samples mentioned above is confirmed. This result will be discussed in Section 3.2.

All parameters of the line shape analyses performed are assembled in Table 2. Firstly, by simulation, we determined the temperature-dependent chemical shifts of samples #1, #3 and #4 in the slow-exchange regime. In that regime, no differences between the chemical shifts of the non-deuterated and the deuterated sample were observed. Therefore, for the line shape calculations of sample #3 in the coalescence region we could use the slow-exchange chemical shifts of sample #4. In spite of the high-field shifts upon increasing temperatures, the signal separation between both OH signals remained almost constant. In addition, the separation did not differ much for the two solvents. The line widths in the absence of exchange were obtained in the slow-exchange regime and extrapolated to higher temperatures. The pseudo-first-order rate constants $k_{\mathrm{ex}}^{\mathrm{HH}}$ were then obtained by adapting the calculated to the experimental spectra. In the fast-exchange region the values are not very precise and were not used later for the discussion. The values of $k_{\mathrm{ex}}^{\mathrm{HD}}$ of sample #4 were obtained in a similar way, but a line shape contribution from the 12% HH species was included. Between 266 and 301 K, the signals were simulated in terms of a one-spin system, which led to larger effective line widths (Table 2).

### 3.2. NMR Chemical Shift Data Analysis

**Figure 11:**
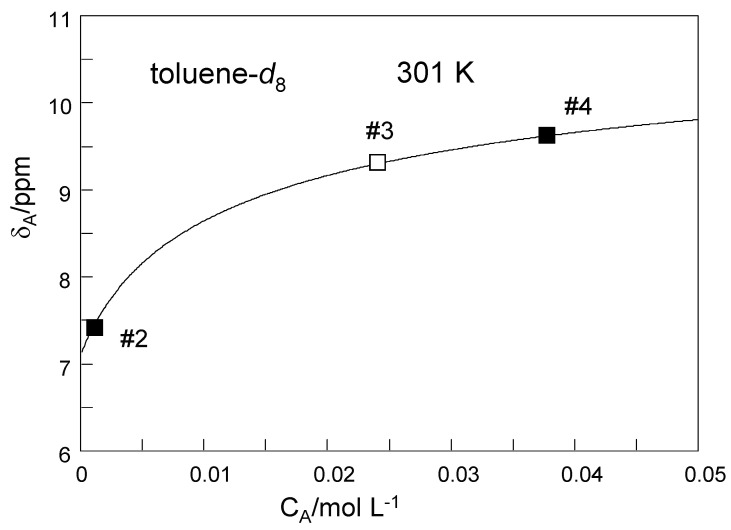
Average ^1^H chemical shift *δ*_A_ of the mobile proton of TMRA as a function of the total concentration C_A_. The solid line was calculated in terms of a dimer–monomer equilibrium using Equation (8). Explanation of symbols: 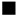: Experimental values of samples #2 and #4 used to determine the parameters of the solid line, i.e., the intrinsic chemical shift of the monomer *δ*_A1_ = 7.1 ppm, of the dimer *δ*_A2_ = 11.5 ppm and of the equilibrium constant *K* = 0.024 mol L^−1^. 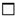: the chemical shift value of sample #3 was placed on the solid line in order to determine the total concentration *C*_A_ = 0.024 mol L^−1^ corresponding to about half of TRMA in the monomer and half in the dimer.

Our first task was to determine the total TMRA concentration *C*_A_ of sample #3. For that purpose, we have plotted in Figure 11 the OH chemical shifts *δ*_A_ of the dilute sample #2 and of the deuterated sample #4 obtained at 301 K as solid squares as a function of the known concentrations C_A_. Then, we reproduced these data using Equation (8) by adapting the monomer chemical shift to a value of *δ*_A1_ = 7.1 ppm and the dimer chemical shift to *δ*_A2_ = 11.5 ppm. The value of *δ*_A2_ is close to the value of 11.34 ppm obtained at low temperatures (Table 2) where only little amounts of monomers are formed. On the other hand the value of *δ*_A1_ is close to the value of 7.4 ppm obtained for the dilute sample #2 at 301 K, containing only small quantities of the dimer. The equilibrium constant *K* was then varied until the best data fit was obtained, leading to a value of *K* = 0.024 mol L^−1^. Finally, we determined the unknown concentration of sample #3 in the following way. We placed an open square symbol in the graph at a value of 9.34 ppm, the OH chemical (Table 2) of sample #3 at 301 K, and moved the symbol horizontally until it was located on the solid line. The abscissa value then provided the unknown concentration of *C*_A_ = 0.024 mol L^−1^of sample #3. This value is almost equal to the value of *K* as expected for a similar number of TMRA molecules present as monomers and in dimers.

**Figure 12:**
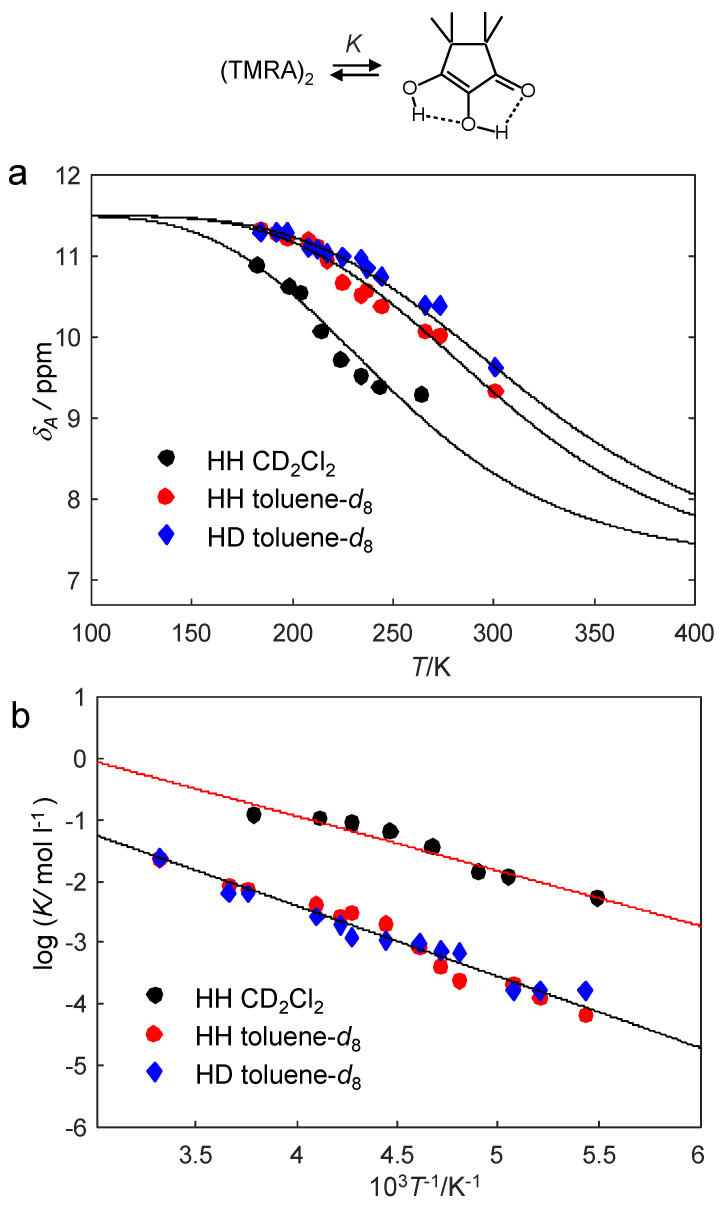
(**a**) Average hydroxyl chemical shifts *δ*_A_ of TMRA dissolved in CD_2_Cl_2_ (sample #1) and in toluene-*d*_8_ (samples #2 and #3) as a function of temperature. The solid lines were calculated in a similar way as those described in Figure 6, but adapted to the experimental data for the determination the dimer–monomer dissociation enthalpies and entropies Δ*H* and Δ*S*. The values of *δ*_A1_ and *δ*_A2_ were the same as in Figure 11 and included in Table 3. (**b**) Van’t Hoff plot of the equilibrium constants *K* of dissociation obtained from the experimental values of *δ*_A_ using Equation (6). For further explanation see text.

In order to be able to construct the Arrhenius diagram of the observed proton exchange of TMRA, we firstly needed to evaluate the information of the dimer–monomer equilibrium from the chemical shift data. For that purpose, we plotted in Figure 12a the average OH chemical shifts *δ*_A_ of TMRA samples #1, #3 and #4 (Table 2) as a function of temperature. The solid lines were calculated in terms of the dimer–monomer equilibrium as described above in the discussion of Figure 6. The parameters used are all assembled in Table 3.

At 184 K, the dimer dominates, exhibiting an intrinsic chemical shift of 11.5 ppm, average of the central and peripheric OH groups. Experimentally, a small quantity of the monomer leads to a small high field shift to 11.34 ppm because of a small quantity of the monomer. At 301 K and low concentrations, the monomer dominates, exhibiting an intrinsic chemical shift of 7.1 ppm (Figure 11). Because of the prescence of some dimer, experimentally a small low field shift to 7.4 ppm is observed. Surprisingly, the solid lines in Figure 12a were obtained with the same limiting values *δ*_A1_ and *δ*_A2_ for the toluene-*d*_8_ and the CD_2_Cl_2_ samples. Moreover, within the margin of error, we could not detect a temperature dependence of these values. The experimental chemical shift differences for the two solvents thus arise almost entirely from the formation of more monomers in the case of CD_2_Cl_2_ as compared to toluene-*d*_8_.

As an alternative to Figure 12a, using the temperature-independent limiting chemical shifts *δ*_A1_ and *δ*_A2_, as well as the temperature-dependent chemical shifts *δ*_A_, wse were able to calculate the monomer mole fractions *x*_A1_ and hence the equilibrium constants *K* using Equations (6) and (7). The values obtained are included in Table 2. The logarithms of the equilibrium constants were then plotted in the van’t Hoff plot of Figure 12b as a function of the inverse temperature. Δ*H* and Δ*S* were obtained by linear regression as illustrated by the solid lines, and then also used to calculate the solid lines in Figure 12a.

### 3.3. Arrhenius Diagrams of the Tautomerism of TMRA

**Figure 13:**
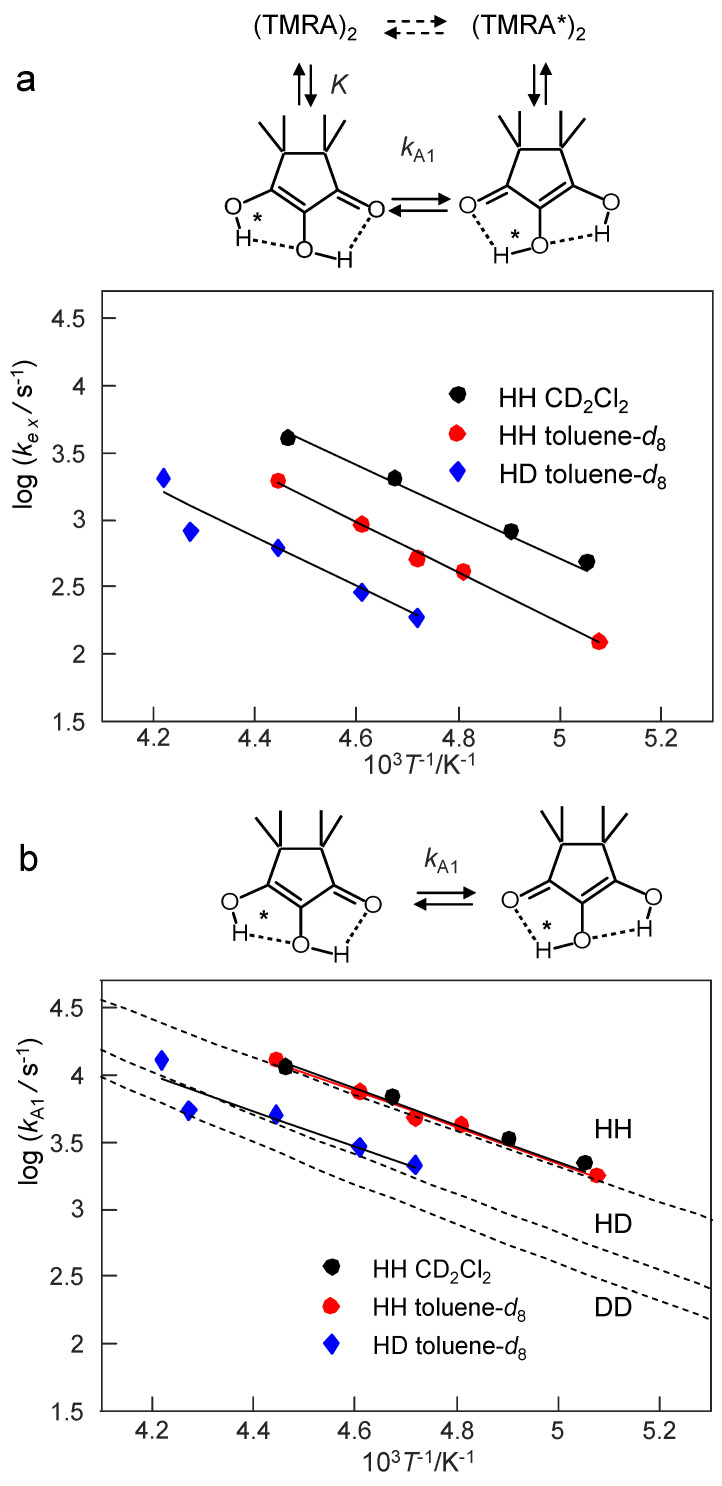
(**a**) Arrhenius diagram of the pseudo-first-order rate constants *k*_ex_ of the tautomerism of TMRA in solution. The values of *k*_ex_ were obtained by line shape analysis (Figure 9, Figure 10 and Figure 11, Table 2) and refer to the temperature-dependent mixture of monomers and dimers. (**b**) Arrhenius diagram of the first-order rate constants *k*_A1_ of the tautomerism of TMRA in solution. The values of *k*_A1_ were obtained from the values of *k*_ex_ using Equation (10). The hatched curves were calculated using the tunneling model described in Section 4.5.

Figure 13 contains two Arrhenius diagrams. Figure 13a consists of a plot of the logarithms of the pseudo-first-order rate constants *k*_ex_ obtained by line shape analysis for the different samples as a function of the inverse temperature. The energies of activation, *E*_aex_ and the logarithms of the pre-exponential factors log *A*_ex_ are included in Table 3. However, these values are difficult to discuss as they refer to a temperature dependent mixture of the monomers and the dimers. Therefore, we calculated the rate constants of tautomerism *k*_A1_ of the monomer alone using Equation (10) from the known equilibrium constants *K*, the total concentrations *C*_A_ and the exchange rate constants *k*_ex_, setting *k*_A2_ ≈ 0. The resulting Arrhenius diagram is depicted in Figure 13b. By linear regression analysis resulting in the solid line, we obtained the activation parameters of the tautomerism of monomeric TMRA, assembled in Table 3. For comparison we added the hatched curves, which were calculated using the tunneling model described in Section 4.5.

## 4. Discussion

### 4.1. General Remarks

We have reported the results of dynamic NMR experiments on tetramethyl reductic acid (TMRA, Figure 3), a vitamin C analogue, dissolved in CD_2_Cl_2_ and in toluene-*d*_8_. The experiments were performed between the years 1984 and 1988, where only one-dimensional variable temperature ^1^H NMR spectroscopy at 90 MHz was available for us. However, the data analysis was entirely updated in 2021, taking into account the progress made in the NMR spectroscopy of hydrogen transfer and bonding.

### 4.2. Solvent-Dependent NMR Spectroscopy and Monomer-Dimer Equilibrium of TMRA

For both toluene-*d*_8_ and CD_2_Cl_2_ as solvents, two OH signals are observed for TMRA at low temperatures, which broaden and coalesce with increasing temperature, indicating a fast exchange of the chemical positions of the peripheric and the central protons. The reaction is substantially faster in CD_2_Cl_2_ as compared to toluene-*d*_8_. At the same time, the OH signals shift to high field when temperature is increasd. The latter phenomenon is explained in a quantitative way in terms of a dimer-monomer equilibrium of TMRA in solution.

Usually, in order to characterize monomer–dimer association equilibria of proton donors, spectra are measured as a function of the total concentration *C*_A_ in order to obtain equilibrium constants as shown for diarylamidines **10** (Figure 2) [21,22]. However, as the preparation of TMRA samples was tedious because of the necessity to exclude air and moisture, and as our resources were limited during the time when the experiments were performed, we decided to concentrate on only a small number of samples and to focus on the temperature rather than the concentration dependence of the NMR spectra of TMRA. As can be inferred from Table 1, for CD_2_Cl_2_ as solvent a single sample (#1) was studied, and for toluene-*d*_8_ a very dilute sample (#2), a concentrated sample (#3), as well as a concentrated sample (#4), where about 90% of the OH groups were deuterated. By line shape analyses the pseudo-first-order rate constants $k_{\mathrm{ex}}^{\mathrm{LL}}$, LL=HH, HD were obtained. The HD transfer was found to be slower than the HH transfer, indicating the presence of kinetic HH/HD isotope effects.

The NMR spectra also indicate that down to 180 K the conversion rates between monomers and dimers are still fast on the NMR timescale. The slow hydrogen bond exchange regime could not be reached here; for that lower temperatures have to be achieved, for example using freons as solvents [7,40,41]. We note that fast hydrogen bond exchange does not lead to an exchange of the central and the peripheric OH protons. That requires multiple proton transfers in the monomer or in the dimers (Figure 5).

In order to obtain the equilibrium constants *K* of the dimer–monomer equilibria, we analyzed the average OH chemical shifts obtained by line shape analysis (Table 2). Using the data analysis of Figure 12, we were able to obtain the monomer mole fractions and the values of *K* as a function of temperature. The *K*-values are substantially larger for CD_2_Cl_2_ as compared to toluene-*d*_8_, indicating that the formation of the monomer is preferred in the polar solvent.

We attribute this finding to the larger dielectric constants of dichloromethane [41] as compared to toluene [42]. By inspection of Figure 5, one can infer that the TMRA monomer exhibits a substantial dipole moment, whereas it is reduced or absent in the dimers. Thus, the monomer is stabilized in the polar solvent, which explains the observed influence of the solvent on the monomer–dimer equilibria.

### 4.3. OH Chemical Shift Assignments and Hydrogen Bond Geometries

In order to assist the OH chemical shift assignment and to obtain information about the hydrogen bond structures of TMRA in solution, we used correlations of OH chemical shifts with OHO hydrogen bond geometries derived from NMR and neutron diffraction data [8]. For the limiting chemical shifts of the two OH groups of the dimer, we obtained the hydrogen bond distances assembled in Table 4. *r*_1_ and *r*_2_ represent the two distances of a H-bonded proton to its neighbors, here, oxygen atoms. For linear hydrogen bonds, *r*_1_ + *r*_2_ is equal to the O…O distance. For a given geometry, the chemical shifts were found to be larger for O-H ··O=C moieties than for O-H ··O-H moieties [8].

The *r*_1_ + *r*_2_ values obtained for the dimer of TMRA in solution are within the margin of error, the same as for the OHO hydrogen bonds of TMRA in the solid state (Figure 4). For the latter, no substantial difference of O…O distances for the O-H ··O=C and O-H ··O-H moieties were observed. Therefore, we tentatively assign the low-field signal of the dimer to the O-H···O=C moiety and the higher-field signal to the O-H···O-H moiety. Unfortunately, we could measure only the average OH chemical shifts of the TMRA monomer, not the individual shifts of the central and the peripheric OH protons. Therefore, we do not have information about its hydrogen bond geometries by NMR measurements. Nevertheless, the high-field shift of the monomer indicates that the intramolecular hydrogen bonds in the monomer are weakened as compared to the dimer.

### 4.4. Arrhenius Diagrams of the TMRA Tautomerism

The observation of two OH signals at 184 K where almost only dimers are present indicates that the tautomerism of the dimers is slow. That indicates that the line broadening and coalescence of the OH signals of TMRA in solution arise from a tautomerism of the monomer.

The corresponding Arrhenius diagrams were depicted in Figure 13. The diagram of Figure 13a depicts the pseudo-first-order rate constants obtained by line shape analysis for the monomer-dimer reaction mixture, whereas the diagram of Figure 13b refers to the monomer of TMRA alone. It was constructed taking into account the equilibrium constants *K*. The activation energies (Table 3) of the monomer are smaller than those of the monomer/dimer mixture; they contain a term arising from the formation of the monomer. Additionally, the pre-exponential factors of the monomer tautomerism are smaller than for the mixture because of the positive reaction entropy of the dissociation of the dimer into the monomers. Thus, the thermodynamic parameters of the equilibrium are eliminated in the activation parameters of the monomer.

Within the margin of error, the rate constants of the tautomerism of the monomer were the same for toluene-*d*_8_ and CD_2_Cl_2_ as solvents (Figure 13b). That means that the observation of a slower proton tautomerism in toluene-*d*_8_ as compared to CD_2_Cl_2_ arises from the fact that the equilibrium is shifted in the latter solvent towards the monomer. This feature was discussed above in terms of a preferential solvation of the polar monomer in the polar solvent.

We note that there is a substantial kinetic HH/HD isotope effect (KIE). It seems that it does not arise from substantially different energies of activation of the HH and the HD transfer, but that the KIE are temperature independent. This statement is, however, tentative in view of the small data basis and the large margin of error of the kinetic data. We return to a discussion of the KIE in the next section.

### 4.5. Mechanism of the TMRA Tautomerism

HH transfer mechanisms are, in principle, multidimensional processes that are difficult to describe theoretically. Nevertheless, for an understanding it is useful to discuss simplified one-dimensional tunneling models such as the Bell–Limbach model [3,4,5,6,19].

**Figure 14:**
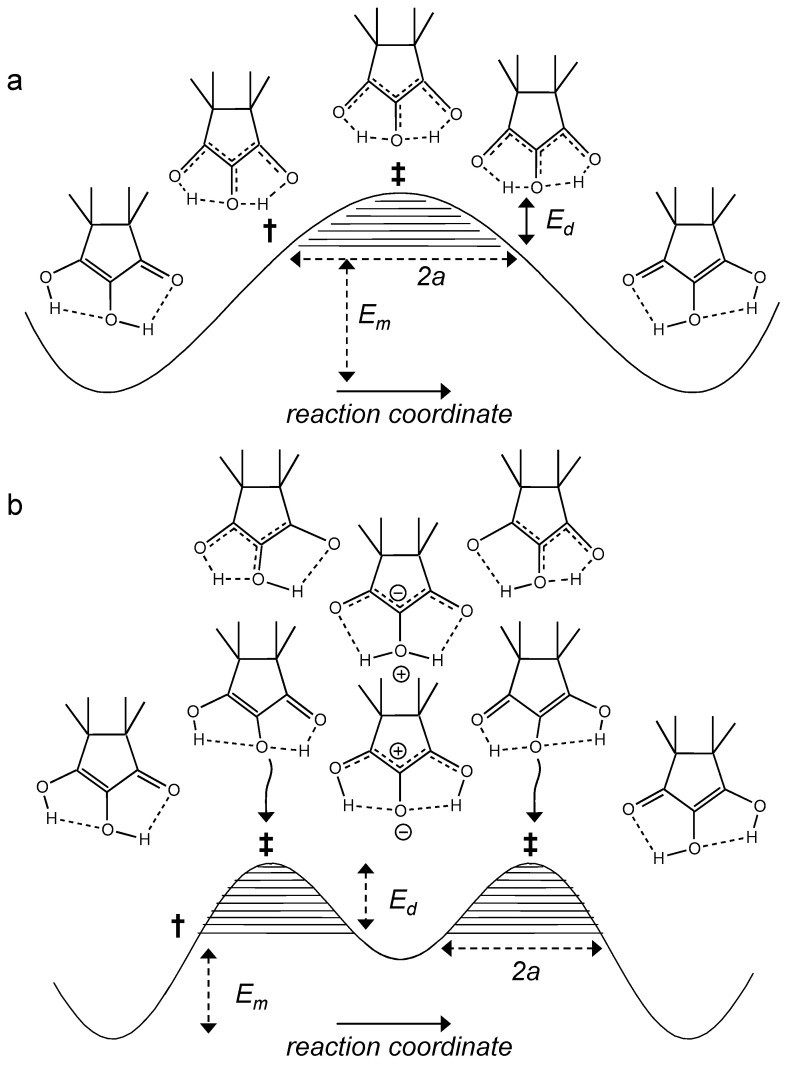
Tautomerization of the TMRA monomer. Hydrogen compression via heavy atom motions requires an energy *E*_m_ to reach the pre-tunneling state **†**. Proton tunneling can occur in the hatched areas below the transition state (energy *E*_m_ + *E*_d_). (**a**) Single barrier tautomerization. Both hydrogen bonds need to be compressed to enable the HH transfer. That requires a large energy for the deformation of the molecular skeleton. (**b**) Double barrier tautomerization of TMRA involving stepwise proton transfers via zwitterionic intermediates. In each step only a single hydrogen bond needs to be compressed, which requires less deformation energy. However, *E*_m_ contains a substantial part arising from the higher energy of the zwitterionic intermediate.

The features of the model are demonstrated in Figure 14. In Figure 14a a single barrier mechanism of a degenerate HH transfer is depicted. H atoms can tunnel through a barrier as the tunneling masses are small. In approximation, the process can be divided in two stages, the compression of both hydrogen bonds and the HH transfer. The compression involves large mass displacements that hinder tunneling between the two potential wells. Only when the system reaches the pre-tunneling state configuration † at energy *E*_m_, tunneling across the remaining barrier of height *E*_d_ can occur in the hatched region. The tunneling masses are then given by

*m* = *m*_H_ + Δ*m* (11)

where *m*_H_ = 2 is the mass of the two hydrogen atoms and Δ*m* is a term arising from small changes of C…O and C…C bond lengths. The larger is the hatched area and the smaller is the tunneling mass, the larger is also the probability of tunneling.

In order to realize a single barrier, the two hydrogen bonds need to be of the cooperative type (Figure 1b), i.e., compression of one bond should lead to the compression of the other bond coupled. That is realized in systems like carboxylic acid dimers or amidines (**7** and **10** in Figure 2) [21,22,23], but it is unlikely for intramolecular hydrogen bonds, as it requires a large energy for the deformation of the molecular skeleton. Intramolecular coupled hydrogen bonds are more often anti-cooperative in the sense that compression of the first bond leads to a widening of the second one [4,5,7]). That leads to a stepwise double proton transfer via an intermediate which is zwitterionic in the case of TMRA, as illustrated in Figure 14b. The minimum energy *E*_m_ to reach the pre-tunneling state now contains a substantial part arising from the higher energy of the intermediate tautomer. There are now two remaining barriers (hatched) along which single H atoms (*m*_H_ = 1) can tunnel.

It has been shown that degenerate HH transfers along single barriers involve two large primary KIE, i.e.,

(12) $$P_{1}=k^{\mathrm{HH}}/k^{\mathrm{HD}},\;P_{2}=k^{\mathrm{HD}}/k^{\mathrm{DD}}$$

where *P*_1_ is mostly a little bit larger than *P*_2_ [5].

By contrast, in the case of a degenerate stepwise HH transfer, in the absence of secondary kinetic isotope effects, there is only a single primary kinetic isotope effect *P*, i.e.,

(13) $$\begin{array}{l} k^{\mathrm{HH}}=k^{H},\\ k^{\mathrm{HD}}=k^{\mathrm{DH}}=\frac{2k^{H}}{1+k^{H}/k^{D}}=\frac{2k^{D}}{1+k^{D}/k^{H}\;},\\ k^{\mathrm{DD}}=k^{D}=k^{\mathrm{HH}}P^{-1}=k^{H}P^{-1}. \end{array}$$

*k*^H^ and *k*^D^ represent the single proton transfer rates. When the kinetic isotope effects *P* are large, the overall rate constants *k*^HD^ are about twice as large as *k*^DD^. Experimentally, that was found for the intramolecular HH transfers in porphyrins **3**, oxalamidines **4** and related systems [5]. We note that secondary KIE’s referring to the replacement of the bound H by D are small.

In order to calculate Arrhenius curves within the Bell–Limbach tunneling model one needs the energies *E*_m_ and *E*_d_ and the tunneling masses as well as the barrier width 2*a* and the pre-exponential factor *A*. It has been shown that for almost all intra- and intermolecular HH transfers *A* is almost independent of the isotope and exhibits values around 10^12.6^ s^−1^. This value is equal to *kT*/*h* expected for a unimolecular reaction around 200 K. Finally, Δ*ε* represents the energy differences of the barriers of the HH and HD, of the HD and DD reactions in the single barrier case and of the H and D reactions in the double barrier case.

**Figure 15:**
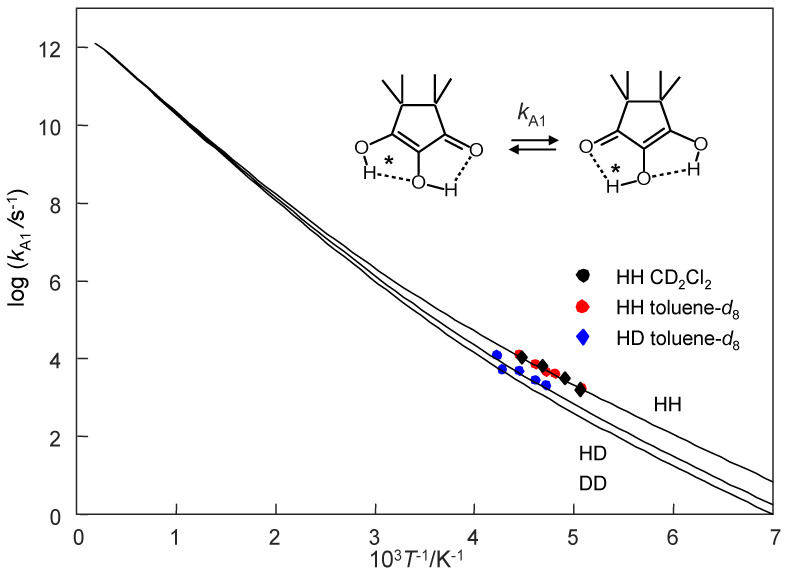
Arrhenius diagram of the tautomerization of TMRA. Plot of log *k* vs. the inverse temperature in the full temperature range. The Arrhenius curves were calculated using the Bell–Limbach tunneling model [3,4,5,6,19] using the parameters of Table 5.

Using this model, we calculated the Arrhenius curves of the tautomerism of monomeric TMRA which are depicted in Figure 15. We only consider here the stepwise two-barrier case. Plotted are the three rate constants defined in Equation (13). The parameters of these curves are assembled in Table 5. The experimental data points are located in the low-temperature region where the curves are parallel, i.e., the kinetic isotope effects are temperature independent. The calculated curves were added as hatched lines to Figure 13b. That explains that the experimental activation energies are very similar and that the pre-exponential factors much smaller than the value of 10^12.6^ s^−1^are used here. The Arrhenius curves are non-linear as is typical for a tunnel mechanism. The tunneling masses of H and D are expected, the additional tunneling mass Δ*m* = 1.5 is similar to the parameters found previously for oxalamidines **4** [5,16].

We note that these parameters might be subject to changes whenever new experiments are performed from which rate constants in a wider experimental range and for the DD reaction are obtained. In addition, such experiments might definitively decide whether the HH transfer in TMRA is a single or a double barrier process.

## 5. Conclusions

Some concluding remarks are as follows.

(i) Using dynamic NMR, we explored the vitamins C analogue tetramethyl reductic acid (TMRA) with respect to its hydrogen bond properties and proton tautomerism in solution. The latter corresponds to a transfer of two H atoms (HH transfer), which interconverts the central and peripheric OH protons. A monomer–dimer equilibrium was established by chemical shift and rate constants of the tautomerism obtained by line shape analysis. The dimer is favored at low temperatures, but when temperature and solvent polarity are raised, the equilibrium is shifted towards the polar monomer. At the same time, the rate constants increase with the monomer concentration. That was in contrast to our expectations, as the hydrogen bonds in the dimer are stronger than in the monomer. A procedure was developed that allowed us to obtain the intrinsic rate constants of the HH and the HD transfer.

(ii) The experiments were performed in the years 1984–1988 when we did not yet have access to multinuclear and multidimensional high-field NMR spectroscopy. However, the experimental data were only analyzed in recent months taking into account the progress made in hydrogen bond NMR spectroscopy. It would be desirable for the future to extend the range of concentration and temperature in order to increase the basis of kinetic and thermodynamic data. In particular, the use of dynamic ^13^C NMR spectroscopy would be helpful to establish the rate constants of the DD transfer as in the case of oxalamidines **4** [16].

(iii) Using liquefied gases as solvents would be interesting to characterize the structures of the dimers in the slow hydrogen bond exchange regime, as has been done for other systems [9,10,11,12].

(iv) Finally, DFT calculations of the reaction pathways and multidimensional tunneling would be important to compare experiment and theory.

## Figures and Tables

**Table 1:** Composition of NMR samples of TMRA studied.

Sample	Solvent	*C*_A_	*X*_D_	*δ*_OH_
#1	CD_2_Cl_2_	0.12 ^a^	0	-
#2	toluene-*d*_8_	0.00108 ^a^	0	7.4
#3	toluene-*d*_8_	0.024 ^b^	0	9.34
#4	toluene-*d*_8_	0.038 ^a^	0.88 ^c^	9.63

^a^ determined by weighing, ^b^ obtained by chemical shift analysis as discussed in Section 3.2. ^c^ determined by integration of the OH vs. the methyl ^1^H signals as described in Section 2.4. Total concentration *C*_A_ of TMRA in mol L^−1^. *δ*_OH_ chemical shift in ppm of averaged OH signals of TMRA at 301 K.

**Table 2:** ^1^H NMR simulation parameters of tetramethyl reductic acid dissolved in CD_2_Cl_2_ and toluene-*d*_8_ in the presence of a degenerate tautomerism.

sample #3, solvent toluene-*d*_8_, deuterium fraction *X*_D_ = 0										
*T*/K	*ν*_1_ − *ν*_TMS_	*ν*_2_ − *ν*_TMS_	*ν*_1_ − *ν*_2_	½(*ν*_1_ + *ν*_2_)	*W*_01_	*W*_02_	*K*/mol L^−1^	*x*_A1_	$k_{\mathrm{ex}}^{\mathrm{HH}}$/s^−1^	$k_{A1}^{\mathrm{HH}}$/s^−1^
184	1179 (13.1)	860 (9.55)	319	1021 (11.34)	15	25	0.6 × 10^−4^	0.05	-	-
192	1174 (13.04)	855 (9.49)	319	1014 (11.28)	5	5	1.2 × 10^−4^	0.07	≈70	-
197	1173 (13.02)	852 (9.46)	321	1012 (11.22)	5	5	2.1 × 10^−4^	0.09	120	1800
208	1160 (12.89)	845 (9.39)	321	1003 (11.14)	5	5	2.4 × 10^−4^	0.09	400	4300
212	1159 (12.87)	841 (9.34)	318	1000 (11.11)	5	5	4.1 × 10^−4^	0.12	500	4800
217	1144 (12.71)	826 (9.18)	318 *	985 (10.95)	5	5	8.5 × 10^−4^	0.16	900	7500
225	1122 (12.46)	807 (8.96)	315 *	964 (10.68)	5	5	2.0 × 10^−3^	0.22	1900	13,000
234	1105 (12.28)	790 (8.78)	315 *	947 (10.52)	5	5	3.0 × 10^−3^	0.25	≈2500	-
237	1110 (12.33)	795 (8.83)	315 *	952 (10.58)	5	5	2.6 × 10^−3^	0.24	≈2800	-
244	1092 (12.13)	777 (6.63)	315 *	934 (10.38)	5	5	4.1 × 10^−3^	0.28	≈4500	-
266	1065 (11.83)	750 (8.33)	315 *	907 (10.08)	5	5	7.3 × 10^−3^	0.33	≈8000	-
273	1060 (11.78)	745 (8.28)	315 *	902 (10.02)	5	5	8.1 × 10^−3^	0.34	≈10,000	-
301	998 (11.09)	683 (7.59)	315 *	841 (9.34)	5	5	2.3 × 10^−2^	0.41	≈15,000	-
sample #4, solvent toluene-*d*_8_, deuterium fraction *X*_D_ = 0.88										
*T*/K	*ν*_1_ − *ν*_TMS_	*ν*_2_ − *ν*_TMS_	*ν*_1_ − *ν*_2_	½(*ν*_1_ + *ν*_2_)	*W*_01_	*W*_02_	*K*/mol L^−1^	*x*_A1_	$k_{\mathrm{ex}}^{\mathrm{HD}}$/s^−1^	$k_{A1}^{\mathrm{HD}}$/s^−1^
184	1179 (13.1)	860 (9.55)	319	1021 (11.34)	20	30	1.6 × 10^−4^	0.06	-	-
192	1174 (13.04)	855 (9.49)	319	1014 (11.28)	20	20	1.6 × 10^−4^	0.06	-	-
197	1170 (13.00)	854 (9.49)	319	1012 (11.24)	5	5	1.6 × 10^−4^	0.06	-	-
208	1160 (12.89)	843 (9.37)	317	1002 (11.13)	5	5	6.8 × 10^−4^	0.12	≈100	-
212	1156 (12.85)	840 (9.33)	317	998 (11.09)	5	5	7.2 × 10^−4^	0.12	180	2130
217	1152 (12.8)	834 (9.26)	318	993 (11.03)	5	5	9.6 × 10^−4^	0.14	280	2900
225	1147 (12.75)	828 (9.25)	315 *	990 (11.0)	5	5	1.1 × 10^−3^	0.15	600	5050
234	1143 (12.73)	831 (9.23)	315 *	988 (10.98)	5	5	1.2 × 10^−3^	0.15	800	5450
237	1138 (12.64)	823 (9.14)	315 *	980 (10.89)	5	5	1.9 × 10^−3^	0.18	2000	12,800
244	1127 (12.53)	812 (9.03)	315 *	970 (10.78)	5	5	2.6 × 10^−3^	0.21	-	-
266				938 (10.42)	37		6.3 × 10^−3^	0.28	-	-
273				935 (10.39)	30		6.4 × 10^−3^	0.28	-	-
301				867 (9.63)	30		2.4 × 10^−2^	0.39	-	-
sample #1, solvent CD_2_Cl_2_, deuterium fraction *X*_D_ = 0										
*T/K*	*ν*_1_ − *ν*_TMS_	*ν*_2_ − *ν*_TMS_	*ν*_1_ − *ν*_2_	½(*ν*_1_ + *ν*_2_)	*W*_01_	*W*_02_	*K*/mol L^−1^	*x*_A1_	$k_{\mathrm{ex}}^{\mathrm{HH}}$/s^−1^	$k_{A1}^{\mathrm{HH}}$/s^−1^
182	1148(12.75)	812 (9.03)	335	980 (10.89)	5	5	0.005	0.18	140	-
198	1124 (12.49)	789 (8.77)	335	957 (10.63)	5	5	0.012	0.23	470	2250
204	1118 (12.41)	783 (8.69)	335 *	950 (10.55)	5	5	0.014	0.25	800	3350
214	1075 (11.94)	740 (8.21)	335 *	907 (10.08)	5	5	0.037	0.33	2000	6800
224	1043 (11.59)	708 (7.87)	335 *	876 (9.73)	5	5	0.065	0.37	4000	11,500
234	1024 (1138)	689 (7.66)	335 *	857 (9.52)	5	5	0.088	0.39	5500	-
243	1012 (11.25)	677 (7.53)	335 *	845 (9.39)	5	5	0.106	0.41	7500	-
264	1044 (11.16)	669 (7.44)	335 *	837 (9.30)	5	5	0.120	0.41	8500	-

* Extrapolated values. *ν_i_* − *ν*_TMS_: chemical shifts in Hz of the OH groups *i* = 1, 2 of TMRA at 90.02 MHz; in parentheses: *δ*_i_ = (*ν_i_* − *ν*_TMS_)/90.02: chemical shifts in ppm; *W*_0*i*_: line widths in the absence of exchange; $k_{\mathrm{ex}}^{\mathrm{LL}}$, LL=HH, HD: pseudo-first order rate constants of interconversion of the central and the peripheric OH groups obtained by line shape analysis (see Figure 8, Figure 9 and Figure 10); *K*: equilibrium constants of dimer- monomer dissociation in mol L^−1^ using the values of Δ*H* and Δ*S* (see Table 3 below); *x*_A1_: mole fraction of the monomer; *k*_A1_: first-order exchange rate constants of the monomer. For further explanation see text.

**Table 3:** Parameters of the tautomerism of TMRA in solution.

Solvent	Toluene-*d*_8_	Toluene-*d*_8_	CD_2_Cl_2_
*C*_A_ concentration of TMRA	0.024	0.038	0.12
*X*_D_ deuteron fraction	0	0.88	0
*δ*_A1_/ppm	7.1	7.1	7.1
*δ*_A2_/ppm	11.5	11.5	11.5
LL isotopes transferred	HH	HD	HH
*E*_aex_/kJ mol^−1^	36.0	35.4	34.0
*log* ($A_{\mathrm{ex}}^{\mathrm{LL}}$/s^−1^)	11.6	11.0	11.5
$k_{\mathrm{ex}}^{\mathrm{HH}}/k_{\mathrm{ex}}^{\mathrm{HD}}$ at 217 K	3.2		-
Δ*H*/kJ mol^−1^ (dimer ⇒ monomer)	22		17
ΔS/J mol^−1^K^−1^ (dimer ⇒ monomer)	42		50
$E_{\mathrm{aA}1}^{\;\mathrm{LL}}$/kJ mol^−1^ (of monomer)	25.6	25.1	26.7
*log* ($A_{A1}^{\mathrm{LL}}$/s^−1^) (of monomer)	10.0	9.5	10.3

Δ*H* and Δ*S*: Enthalpy and entropy of the dissociation of the TMRA dimer into the monomers. *δ*_A1_ and *δ*_A2_: limiting chemical shifts averaged over both OH groups of the monomer and the dimer. *E*_aex_ experimental energy of activation and $A_{\mathrm{ex}}^{\mathrm{LL}}$ experimental pre-exponential factor of the tautomerism of TMRA. $E_{\mathrm{aA}1}^{\;\mathrm{LL}}$ energy of activation and $A_{A1}^{\mathrm{LL}}$ pre-exponential factor of the tautomerism of the TMRA monomer.

**Table 4:** Estimates of hydrogen bond geometries of the TMRA dimer from the OH chemical shifts.

		*δ*/ppm	½(*r*_1_ − *r*_2_)/Å	*r*_1_ + *r*_2_ /Å	*r*_1_/Å	*r*_2_/Å	*r*_O…O_/Å
dimer	O-H···O=C	13.1 ^a^	0.34 ^b^	2.64 ^b^	0.98 ^b^	1.66 ^b^	2.63
dimer	OH···OH	9.55 ^a^	0.36 ^b^	2.68 ^b^	0.98 ^b^	1.70 ^b^	

*r*_1_ and *r*_2_ are the O-H and H···O distances. ^a^ Sample #3 at 184 K (Table 2). ^b^ Calculated using the correlation of OH chemical shifts vs. ½(*r*_1_ − *r*_2_) [8]. *r*_O…O_: average O…O distances from the X-ray structure of TMRA [33] (see Figure 4).

**Table 5:** Bell-Limbach tunneling model parameters of the tautomerism of TMRA.

Solvent	Toluene-*d*_8_	Toluene-*d*_8_	CD_2_Cl_2_
	HH	HD	HH
*E*_m_/kJmol^−1^	21.8	22.6	21.8
*E*_d_/kJmol^−1^	22.6	20.1	22.6
*log* (*A*/s^−1^)	12.6	12.6	12.6
*m*_H_/a.m.u.	1	1	1
*m*_D_/a.m.u.	2	2	2
Δ*m*/a.m.u.	1.5	1.5	1.5
2*a*/Å	0.18	0.18	0.18
Δ*ε*/kJmol^−1^	0.8		-

## Data Availability

Not applicable.

## Sample Availability

Samples of the compounds are not available from the authors.
